# Enhanced immunogenicity of CTL antigens through mutation of the CD8 binding MHC class I invariant region

**DOI:** 10.1002/eji.200636765

**Published:** 2007-05

**Authors:** Linda Wooldridge, Anna Lissina, Jonathan Vernazza, Emma Gostick, Bruno Laugel, Sarah L Hutchinson, Fareed Mirza, P Rod Dunbar, Jonathan M Boulter, Meir Glick, Vincenzo Cerundolo, Hugo A van den Berg, David A Price, Andrew K Sewell

**Affiliations:** 1 Department of Medical Biochemistry & Immunology, University of CardiffCardiff, UK; 2 Peter Medawar Building for Pathogen Research, University of OxfordOxford, UK; 3 Human Immunology Unit, Weatherall Institute of Molecular Medicine, John Radcliffe HospitalOxford, UK; 4 Novartis Institute for Biomedical ResearchCambridge, USA; 5 Institute of Mathematics, University of WarwickCoventry, UK

**Keywords:** CD8, Cytotoxic T cells, MHC class I, T cell activation, Tumor immunity

## Abstract

CD8^+^ cytotoxic T lymphocytes (CTL) are key determinants of immunity to intracellular pathogens and neoplastic cells. Recognition of specific antigens in the form of peptide-MHC class I complexes (pMHCI) presented on the target cell surface is mediated by T cell receptor (TCR) engagement. The CD8 coreceptor binds to invariant domains of pMHCI and facilitates antigen recognition. Here, we investigate the biological effects of a Q115E substitution in the α2 domain of human leukocyte antigen (HLA)-A*0201 that enhances CD8 binding by ∼50% without altering TCR/pMHCI interactions. Soluble and cell surface-expressed forms of Q115E HLA-A*0201 exhibit enhanced recognition by CTL without loss of specificity. These CD8-enhanced antigens induce greater CD3 ζ chain phosphorylation in cognate CTL leading to substantial increases in cytokine production, proliferation and priming of naive T cells. This effect provides a fundamental new mechanism with which to enhance cellular immunity to specific T cell antigens.

## Introduction

The TCR coreceptor CD8 binds to largely invariant domains of MHC class I (MHCI) 1 and facilitates the process of antigen recognition by a number of mechanisms 2–4. To date, it has been established that CD8 aids antigen recognition by recruiting the TCR to specific membrane domains believed to be privileged sites for TCR-mediated signal transduction 5, recruiting essential signalling molecules to the intracellular side of the TCR/CD3/ζ complex 6, 7 and stabilising the TCR/peptide-MHCI complex (pMHCI) interaction at the cell surface 8, 9. It is not yet clear whether CD8 has other roles in antigen recognition.

The role of CD8 has traditionally been examined using transfected T cell hybridomas or anti-CD8 antibodies. These reagents do not enable discrimination between the role of the pMHCI/CD8 interaction and other possible roles of CD8 in antigen recognition such as direct coupling of CD8 to the TCR 10, 11. MHCI mutations that reduce or abrogate the pMHCI/CD8 interaction without affecting TCR binding 6, 10, 12–15 have enabled the study of this interaction in isolation. The T cell coreceptors CD8 and CD4 play a unique role in biology by assisting in the recognition of pMHCI and pMHCII antigens, respectively, without altering the specificity of TCR/pMHC interactions. This role is facilitated by the uniquely low affinities of the pMHCI/CD8 and pMHCII/CD4 interactions. Human CD8 and CD4 bind to their respective pMHC ligands with K_D_ >100 μM and represent the very weakest 1:1 molecular interactions at the cell surface to which a biological function can be attributed 16.

Previous studies have compared the recognition of antigen in the presence or absence of wild-type pMHCI/CD8 interactions 6, 12–15, 17–20. In addition, a recent study has examined the normalisation of the non-canonical HLA-A*6801 CD8 binding domain 21. However, the biological effects of supranormal CD8 binding have not been examined before. Here, we undertake the first study of the biological effects of increasing the affinity of the pMHCI/CD8 interaction using mutations that enhance CD8 binding while TCR/pMHCI interactions remain faithful.

## Results

### Enhancement of the HLA-A2/CD8 interaction

We have recently characterized a series of mutations in the CD8 binding domain of HLA-A*0201 (HLA-A2 from hereon) that alter the binding of CD8αα, determined by surface plasmon resonance (SPR), without changing the interaction with HLA-A2-restricted TCR. The CD8 interaction with pMHCI is dominated by CD8α 22. CD8αα and CD8αβ bind to pMHCI with similar affinities 23, 24. Mathematical modeling predicts a linear relationship between the monomeric affinity of the HLA-A2/CD8 interaction and TCR/pMHCI off rate. Consistent with this, we observe a linear relationship between the monomeric affinity of HLA-A2/CD8αα binding determined by SPR and TCR/pMHCI off rate on the cell surface of CTL which express CD8αβ. This suggests that our MHCI mutated molecules bind to human CD8αα and CD8αβ with similar affinities 9.

**Figure 1:**
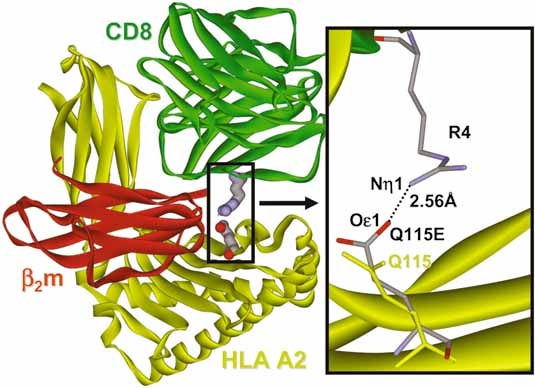
The CD8α1 R_4_ guanidinium moiety forms a stronger electrostatic interaction with the HLA-A*0201 Q115E mutant carboxylate than with the wild-type Q residue. HLA-A2 heavy chain is shown in yellow complexed with β2m (red) and CD8 (green). The inset shows the location of the key residues Q_115_ in HLA-A2 and R_4_ in CD8α1. For clarity, the right-hand-side close up is rotated about the vertical relative to the standard view. The wild-type Q_115_ residue is shown for comparison (yellow). The Q_115_/R_4_ Oε1..Νη1 distance in the HLA-A2 CD8αα crystal structure 1 is 3.18 Å. This distance is predicted by the molecular dynamics simulation to shorten to an average of 2.56 Å with Q_115_E (shown as broken line) and enables these moieties to form a strong electrostatic interaction.

We have further used a range of biophysically characterized altered peptide ligands to show that the about two-fold stabilisation of TCR/pMHCI interactions afforded by CD8 binding 9 remains constant over the entire range of TCR/pMHCI interactions that we are able to study at the cell surface in the absence of CD8 binding (K_D_ 2–40 μM) (Laugel *et al.*, unpublished). Molecular dynamics studies predicted that a Q_115_-to-E (Q115E) mutation in the α2 domain of HLA-A2 would shorten a key hydrogen bond and enhance CD8 binding (Fig. 1). SPR studies confirmed that Q115E HLA-A2 exhibited enhanced CD8 binding without affecting the binding of cognate TCR 9. Thus, the Q115E mutation generates HLA-A2 molecules for which CD8 interaction is enhanced by ∼50% (K_D_ ∼85 μM as compared to K_D_ ∼130 μM for the wild-type interaction) 9. Here, for the first time, we examine the biological consequences of enhanced CD8 binding using this MHCI mutation.

### CD8 enhancement results in improved antigen recognition at the cell surface

**Figure 2:**
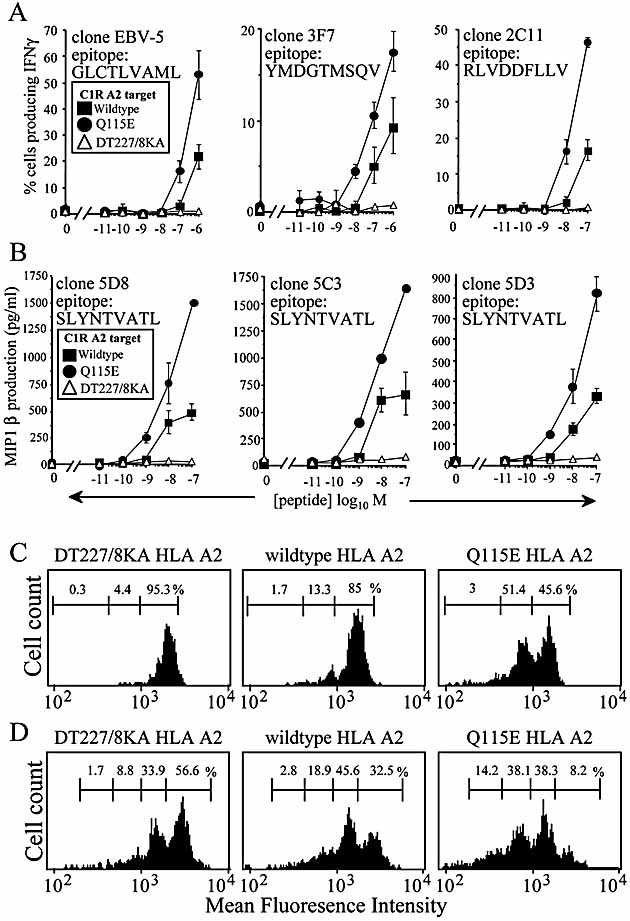
CTL antigen sensitivity and effector function are enhanced by a small increase in the HLA-A2/CD8 interaction. (A) Number of spots produced in IFN-γ ELISPOT with EBV-, tyrosinase- or telomerase-specific HLA-A2-restricted CTL clones in response to peptide-pulsed C1R target cells expressing wild-type HLA-A2, DT227/8KA HLA-A2, or Q115E HLA-A2. Assays were performed with 2.5×10^2^ CTL and 2.5×10^4^ C1R transfectants at 37°C for 4 h. (B) MIP-1β production by a panel of HIV-1 Gag-specific HLA-A2-restricted CTL clones in response to the above-mentioned C1R targets. CTL (2.5×10^4^) were incubated with 2.5×10^4^ C1R transfectants at 37°C for 4 h. Supernatant was assayed by chemokine ELISA as previously described 44. (C, D) Proliferation of the CFSE-labelled anti-tumour CTL clones 1C2 (C) and ILA1 (D), specific for the HLA-A2-restricted telomerase-derived epitopes RLVDDFLLV and ILAKFLHWL, respectively, stimulated with the indicated C1R targets pulsed with 1 μM cognate peptide. Proliferation results over 6 days are indicated by progressive dilution of cellular CSFE content; each division can be visualized incrementally and quantified as a percentage of the total population as depicted.

Antigenic peptide presented in the context of Q115E HLA-A2 is recognized substantially better than in the context of wild-type HLA-A2 (Fig. 2). We recently used the series of HLA-A2 molecules mentioned above that vary in their CD8 binding by >1000-fold while retaining faithful interactions with HLA-A2-specific TCR to model the contribution that CD8 makes to TCR/pMHCI stabilisation at the cell surface 9. These data show that the increased affinity for CD8 afforded by the Q115E substitution in HLA-A2 extends the mean duration of the TCR/pMHCI interaction by only <2% 9. This small enhancement of TCR/pMHCI stability would, by itself, not be expected to improve CTL activation by target cells to the levels observed in a variety of functional recognition assays (Fig. 2). These findings strongly suggest that stabilisation of the TCR/pMHCI interaction is not the sole, and indeed not the major, mechanism through which the CD8 coreceptor enhances ligand recognition.

**Figure 3:**
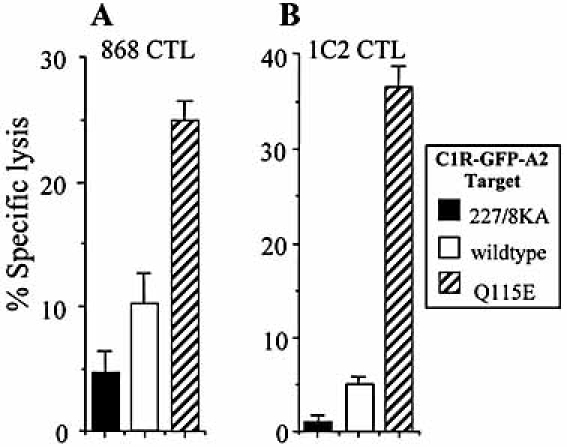
A small enhancement in the HLA-A2/CD8 interaction significantly improves CTL-mediated lysis. C1R cells expressing identical levels of GFP-DT227/8KA HLA-A2, GFP-wild-type HLA-A2 or GFP-Q115E HLA-A2 (Fig. 4) were pulsed for 1 h with 1 μM SLYNTVATL peptide (A) or 1 μM RLVDDFLLV peptide (B). C1R target cells (1.5×10^4^) were incubated with 3×10^4^ cognate CTL in a 96-well U-bottomed plate for 2 h. Specific lysis was calculated by counting CIR-GFP-A2 cells using flow cytometry. Background lysis in the absence of peptide was near zero at a 2:1 E:T ratio. The error bars indicate the standard error from the mean of two replicate experiments.

Antigen presented in the context of Q115E HLA-A2 was shown to be better at inducing IFN-γ and MIP-1β production from a total of 14 different CTL clones specific for seven different antigens (Fig. 2A, B and data not shown). Q115E HLA-A2-expressing cells were also found to be superior targets in a CTL lysis assay (Fig. 3). Notably, antigen-presenting cells (APC) bearing peptide antigen in the context of Q115E HLA-A2 were able to induce substantially better proliferation of tumour-specific CTL clones specific for several different epitopes (Fig. 2C, D and data not shown).

**Figure 4:**
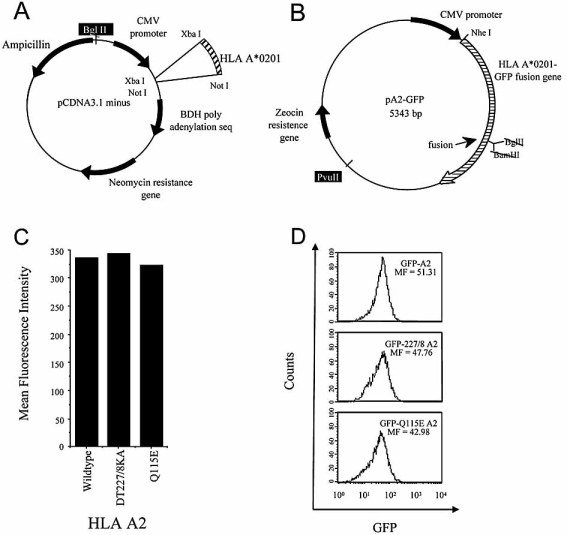
HLA-A2 and GFP-HLA-A2 vectors used in this study and cell surface expression of HLA-A2. (A) HLA-A2 and HLA-A2 mutants were expressed as full-length molecules in pCDNA3.1 (Invitrogen) under G418 (Neo) selection. (B) HLA-A2-GFP fusion constructs were expressed in pGFP-N2 (Perkin Elmer) under Zeo selection. Vectors were linearized by cutting with BglII or PvuII (highlighted) prior to transfection. (C) C1R cells were transfected with wild-type HLA-A2, DT227/8KA HLA-A2 or Q115E HLA-A2; vectors are shown in (A). Transfectants were grown up from single clones and clones with similar expression levels of HLA-A2 were selected. Cells (2.5×10^5^) in 100 μL of FACS buffer were stained with 2.5 μL of the HLA-A2-specific antibody BB7.2 conjugated with FITC (Serotec) for 20 min, washed twice in FACS buffer and analysed on a FACSCalibur (BD Biosciences). Bars show average mean fluorescence intensity of two experiments performed 2 wk apart. (D) C1R targets were transfected with HLA-A2-GFP fusion constructs in the vector shown in (B) and cloned by limiting dilution. Clones were analysed for GFP expression by FACS. Mean fluorescence is shown for clones used as targets in activation assays.

We excluded the possibility that these differences were due to small differences in HLA-A2 density on the APC surface by examining multiple C1R-A2 clones with identical levels of HLA-A2 surface expression as determined by staining with an HLA-A2-specific monoclonal antibody (BB7.2) (Fig. 4C). In addition, we manufactured GFP fusion vectors of all the HLA-A2 mutants used in this study (Fig. 4B). These targets express identical levels of GFP by flow cytometry (Fig. 4D) and fluorescence microscopy confirmed that the vast majority of this protein is associated with the cytoplasmic membrane (data not shown). Target cells expressing C1R-A2 or C1R-GFP-A2 gave similar results in functional T cell assays and clearly demonstrate that the Q115E mutation in HLA-A2 results in substantial improvements in antigen recognition by cognate CTL.

### An incremental increase in HLA-A2/CD8 affinity enhances CTL priming

**Figure 5:**
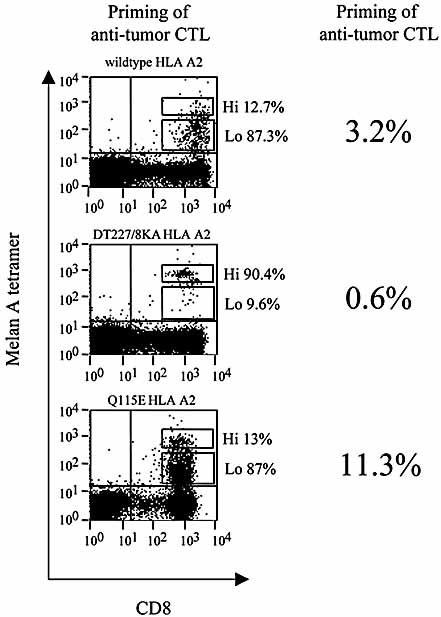
An incremental increase in HLA-A2/CD8 affinity enhances CTL priming. Fresh HLA-A2^+^ human PBMC (10^6^) were incubated with peptide-pulsed, irradiated C1R cells as indicated at an E:T ratio of 5:1. Melan A tetramer^+^ CD8^+^ cells were not detected at day 0 (data not shown). Plots show live cells staining with tetramer and anti-CD8 antibody on day 10 post-priming. Similar results were obtained with the PBMC from four other HLA-A2^+^ individuals in a total of six experiments (data not shown). Targets expressing Q115E HLA-A2 consistently ‘primed’ three to five times more Melan A-specific CTL than targets expressing wild-type HLA-A2. Targets expressing DT227/8KA HLA-A2 only primed Melan A-specific CTL as shown above in two of six experiments.

Thymic output in healthy HLA-A2^+^ individuals is known to generate a high frequency of naive CD8^+^ T cells that can recognize the self antigen Melan A/MART1 25. This system represents the only known naive self-peptide-specific T cell repertoire directly accessible in humans 25. We have previously examined the priming of naive CTL directly *ex vivo* 26. Here, we used this system to show that antigen presented in the context of Q115E-substituted HLA-A2 can prime substantially more Melan A tetramer^+^ CD8^+^ cells from HLA-A2^+^ peripheral blood mononuclear cells (PBMC) than wild-type antigen (Fig. 5). Similar results were observed with HLA-A2^+^ PBMC from four other individuals in six separate experiments (data not shown). Cells bearing antigen in the context of CD8-null (DT227/8KA) HLA-A2 consistently primed far fewer CTL than targets bearing wild-type antigen. In four of six experiments, CD8-null targets failed to prime any Melan A-specific CTL at all (data not shown).

Peptide-pulsed wild-type HLA-A2 target cells induced a tetramer^+^ population with a heterogeneous staining pattern that could be arbitrarily split into tetramer^high^ and tetramer^low^ populations previously shown to correspond to high- and low-avidity CTL, respectively 27. In donors where CD8-null targets were able to prime Melan A-specific CTL, it is notable that only tetramer^high^ CTL emerged (Fig. 5). This is consistent with a recent study 28 that used mice transgenic for HLA-A2, which does not bind to murine CD8 6, to generate high-affinity TCR specific for an epitope from p53. Importantly, Q115E HLA-A2 targets not only induced three to five times more Melan A-specific CTL than wild-type HLA-A2 targets but also primed a large proportion of tetramer^high^ CTL. High-avidity CTL are thought to be optimal for adoptive CTL transfer therapy as they have a proven ability to control tumour growth *in vivo*. Tools such as Q115E HLA-A2 target cells that have the potential to expand CTL with a high avidity for pMHCI from PBMC could therefore facilitate the development of adoptive CTL transfer therapies.

### An incremental increase in HLA-A2/CD8 affinity induces better ζ chain phosphorylation

**Figure 6:**
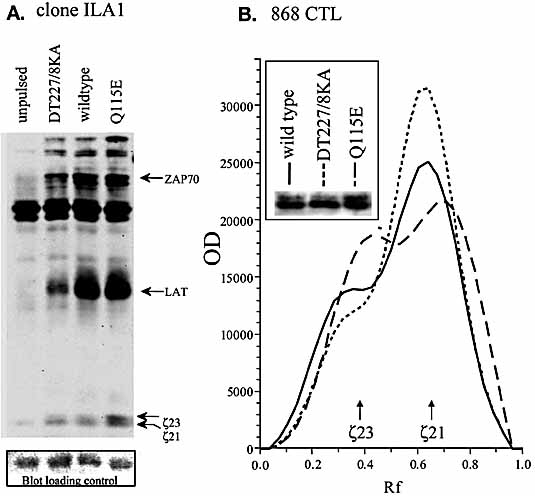
Enhancing the pMHCI/CD8 interaction increases early TCR ζ chain phosphorylation. One million HLA-A2-restricted, telomerase-specific CTL of clone ILA1 (A) and 2×10^6^ HLA-A2-restricted HIV-1 Gag-specific CTL of clone 868 33 (B) were exposed to peptide-pulsed C1R targets bearing the indicated HLA-A2 molecules for 10 min at an E:T ratio of 10:1. Cell extracts were run by SDS-PAGE and blotted with anti-phosphotyrosine antibody as described previously 18. (A) An entire blot is shown. (B) Detail of partially phosphorylated ζ21 and fully phosphoylated ζ23. Chemiluminescent signal was collected with a Bio-Rad Fluor-S™ Multimager. Retardation factor (Rf) is shown *vs.* chemiluminescent intensity for proteins in the 20–24 kD range; a digital image of the corresponding blot is shown inset, with a key to line traces.

To investigate the mechanisms underlying the enhanced antigenicity of Q115E-substituted antigens, we examined the early intracellular signalling events induced by wild-type and CD8-enhanced (Q115E) HLA-A2 ligands and compared this to CD8-null (DT227/8KA) ligands. CD8-enhanced HLA-A2 induced identical levels of tyrosine phosphorylation to wild-type HLA-A2 for most proteins (Fig. 6A) with one notable exception. C1R targets bearing Q115E HLA-A2 were able to induce more of the fully phosphorylated p23 form 29 of the TCR ζ chain in anti-tumour and anti-viral CTL compared to similar targets bearing wild-type HLA-A2 standardized for surface antigen expression levels (Fig. 6A, B). This suggests that the improved antigenicity of CD8-enhanced antigen may be afforded by an increased ability of CD8-associated p56^lck^ to phosphorylate the cytoplasmic immunoreceptor tyrosine activation motifs (ITAM) of the TCR/CD3 complex.

It is curious that we did not observe increased tyrosine phosphorylation of other proteins thought to be further down the TCR-mediated signal transduction cascade, such as ZAP70, with CD8-enhanced antigen. This suggests that the phosphorylation of these downstream proteins may not require full phosphorylation of large amounts of the TCR ζ chain. However, we cannot exclude the possibility that there are differences at other time points. These findings also suggest that the enhanced recognition of Q115E antigens may be channelled through non-protein tyrosine kinase events downstream of ζ chain phosphorylation.

### Incremental CD8 enhancement does not affect T cell specificity substantially

**Figure 7:**
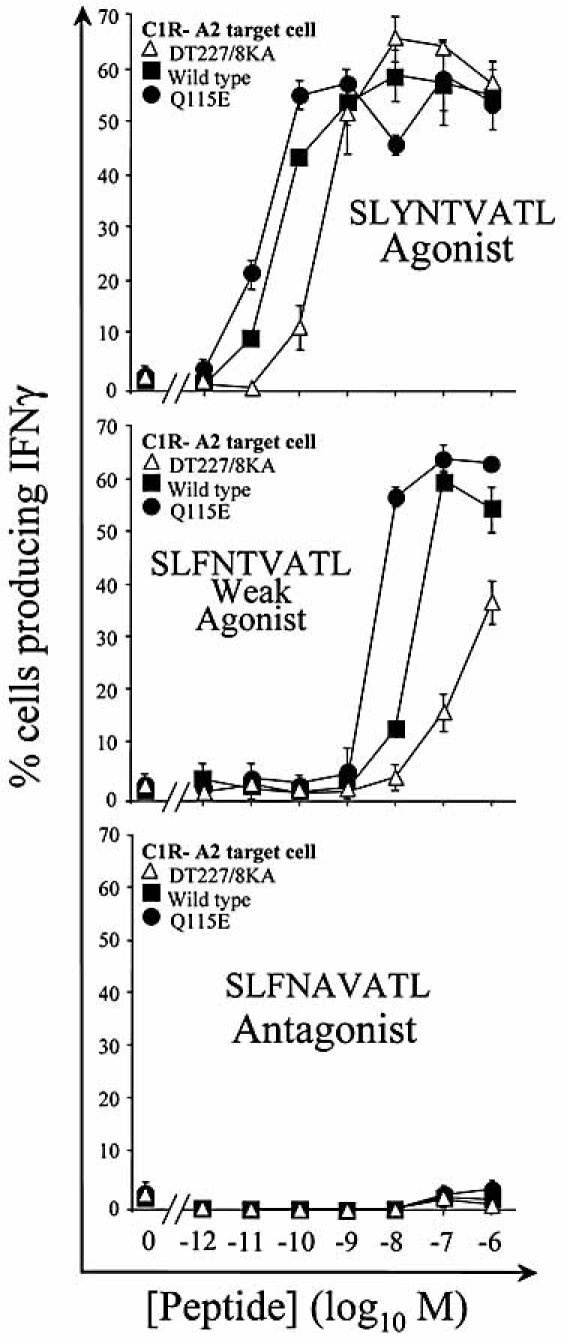
Increasing the pMHCI/CD8 interaction does not affect the specificity of CTL recognition when peptide is presented on the cell surface. HIV-1 Gag-specific CTL (10^3^) of line 868 were incubated with C1R target cells expressing wild-type HLA-A2, DT227/8KA HLA-A2, or Q115E HLA-A2 bearing naturally occurring variants of the SLYNTVATL peptide 33 in an IFN-γ ELISPOT assay 18. In all conditions of activation, 25% of line 868 were tetramer^+^. Results are plotted as % of tetramer^+^ cells producing a spot in the assay. The SL**F**N**A**VATL peptide acts as a strict antagonist of these cells 33, 45.

Importantly, a small increase in the affinity of the pMHCI/CD8 interaction did not result in significant loss of antigen specificity. Enhanced recognition of Q115E-substituted HLA-A2 extends to weak agonist ligands but does not result in enhanced recognition of weaker ligands such as antagonist and null peptides that are known to have a significantly shorter TCR/pMHC mean dwell time 30–32 (Fig. 7). We have previously determined that the 3F/5A variant of the HIV-1 Gag-derived epitope SLYNTVATL (residues 77–85) acts as a strict antagonist of 868 CTL 33 in that it disrupts agonist-induced TCR-mediated signal transduction 6 without itself inducing functional responses. Ligands that act in this manner are believed to have shorter TCR/pMHC mean dwell times 34, 35. Q115E-substituted HLA-A2 was able to augment responses to agonist and weak agonist ligands but did not turn an antagonist ligand into an agonist (Fig. 7 and data not shown).

The pattern of enhancement in ligand recognition we observe is in agreement with studies examining the role of the CD4 coreceptor 31, 32. Thus, our data fit with the ‘sequential engagement’ model of TCR/coreceptor operation, in which the coreceptor acts as a molecular timer and is only able to contribute to ligand recognition if the TCR/pMHCI interaction is of sufficient duration 31, 32. Fluorescence resonance energy transfer experiments lend some credence to this hypothesis by showing that agonist, but not antagonist, ligands trigger intermolecular interaction between CD4 and the TCR 36.

### Enhanced recognition of Q115E-substituted HLA-A2 antigens extends to soluble molecules

**Figure 8:**
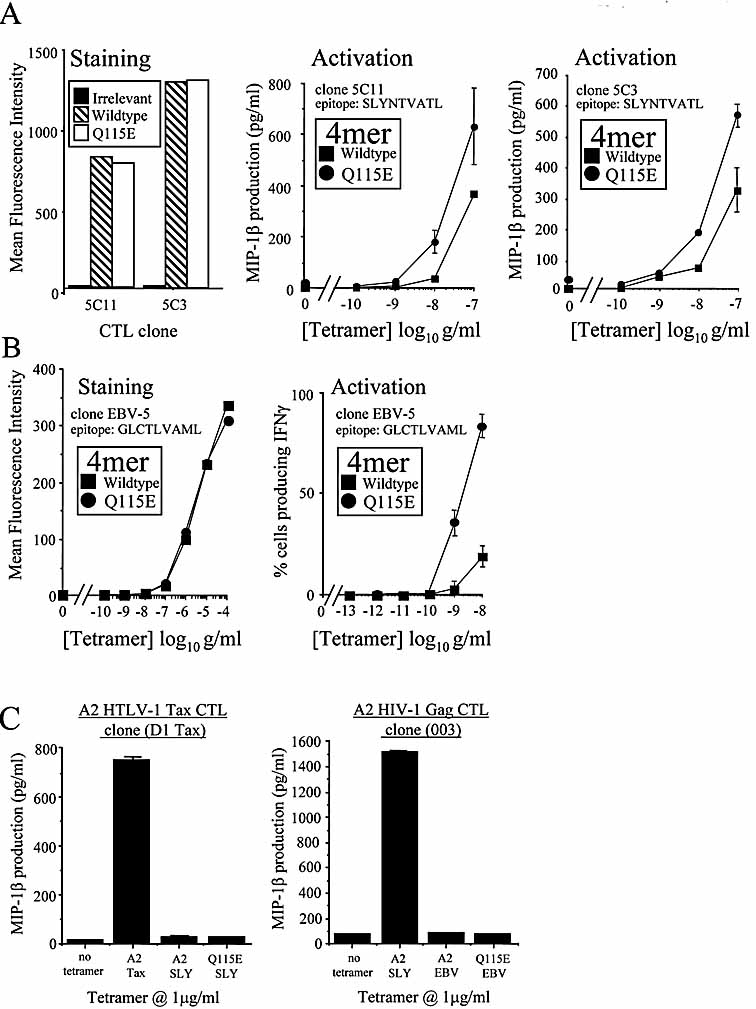
Soluble pMHCI tetramers with increased affinity for CD8 enhance CTL activation. (A, B) 10^5^ HLA-A2-restricted HIV-1 Gag-specific CTL of clones 5C11 and 5C3 (A), or HLA-A2-restricted EBV-specific CTL of clone EBV-5 (B), were stained with 1 μg of either wild-type HLA-A2 or Q115E HLA-A2 tetramer bearing the relevant cognate peptides for 30 min at 37°C in 20 μL PBS. For functional activation assays, (A) 2.5×10^4^ 5C11 or 5C3 cells were incubated with either HLA-A2-SLYNTVATL or Q115E HLA-A2-SLYNTVATL tetramer at the concentrations indicated for 4 h at 37°C, and supernatant was assayed for MIP-1β; (B) 2×10^3^ HLA-A2-restricted EBV-specific CTL were incubated with either HLA-A2-GLCTLVAML or Q115E HLA-A2-GLCTLVAML tetramer for 4 h in an IFN-γ ELISPOT assay. Staining profiles with each tetramer are shown for comparison in the left panel. (C) HLA-A2 HTLV-1 Tax_11–19_-specific CTL (clone D1; 2.5×10^4^) or HLA-A2-restricted HIV-1 p17 Gag-specific CTL (clone 003; 2.5×10^4^) were incubated with tetramers as shown at 1 μg/mL overnight at 37°C. Supernatant was assayed for MIP-1β by ELISA as described previously 44.

**Figure 9:**
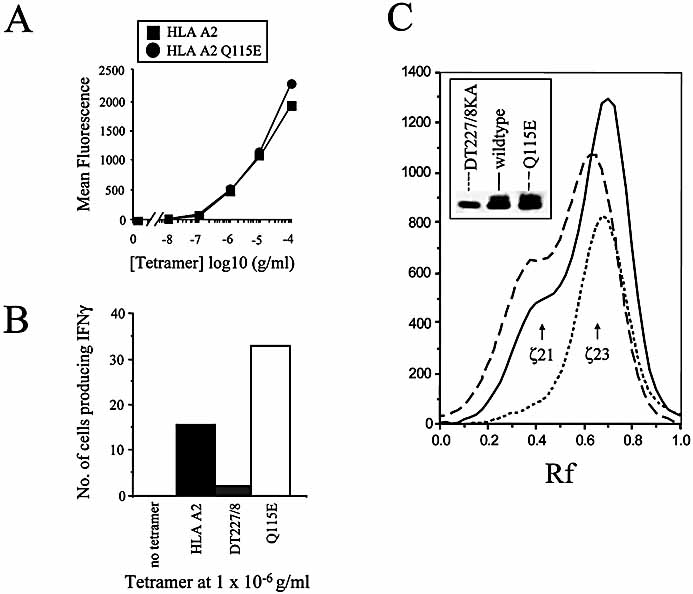
Tyrosine phosphorylation of the CD3 ζ chain is enhanced by increasing the affinity of soluble pMHCI antigen for CD8. (A) HIV-Gag specific CTL (10^5^) of clone 003 were stained in 20 μL of PBS with indicated concentrations of either wild-type HLA-A2-SLYNTVATL or Q115E HLA-A2-SLYNTVATL tetramer for 30 min at 37°C. (B) HLA-A2-restricted HIV-1 p17 Gag-specific CTL (5×10^2^) were incubated with indicated cognate tetramers for 4 h in an IFN-γ ELISPOT assay. (C) Clone 003 CTL (10^6^) were exposed to 10 μg/mL of indicated cognate HLA-A2 tetramer for 3 min and cell extracts were run by SDS-PAGE and blotted with anti-phosphotyrosine antibody as described previously 18. Retardation factor (Rf) is shown *vs.* chemiluminescent intensity for proteins in the 20–24 kD range; a digital image of the corresponding blot is shown inset as for Fig. 6B.

Finally, we examined whether increased recognition of CD8-enhanced antigens could extend to soluble ligands. Tetrameric pMHCI complexes have revolutionized the study of antigen-specific T cells 37, 38 and have been used to study the activation requirements of CTL without the need for an APC 6, 39. Tetramerized Q115E-substituted HLA-A2 bound to cell surface TCR of cognate CTL with similar affinity to wild-type HLA-A2 tetramers when analysed following a 30-min incubation (Fig. 8, 9, 9 and data not shown). Despite the identical cross-linking properties that were observed at 30 min, tetrameric forms of Q115E-substituted HLA-A2 elicited IFN-γ and MIP-1β secretion from cognate CTL in greater quantities and in response to lower concentrations of soluble antigen compared to wild-type HLA-A2 4 h post-stimulation (Fig. 8A, B). This increased activation applied to only those CTL bearing a cognate TCR (Fig. 8C). Similar results were obtained for the anti-HIV-1 Gag CTL clone 003 (Fig. 9A, B). Interestingly, HLA Q115E-substituted reagents induced better tyrosine phosphorylation of the TCR ζ chain (Fig. 9C). Thus, as for cell surface-presented antigen (Fig. 6), the increased immunogenicity of Q115E-substituted HLA-A2 in soluble form appears to be the result of enhanced early CD8-mediated signal transduction (Fig. 9)

## Discussion

Recent studies of murine TCR with variable affinity in the presence or absence of CD8 indicate that, in order to elicit CTL activation, virtually all normal syngeneic interactions require this coreceptor 17. Furthermore, CD8 is essential for formation of the immunological synapse, a requirement that cannot be overcome by increasing antigen concentration 40. Thus, CD8 plays an essential role in the physiological recognition of MHCI-restricted peptide antigens by CTL. This role for CD8 is mediated through at least three mechanisms: (1) stabilisation of the TCR/pMHCI interaction at the cell surface by approximately two-fold 9; (2) topographical organization of cell surface TCR 5; and (3) recruitment of intracellular signalling molecules to the cytoplasmic side of the TCR/CD3/ζ complex. Most of the CD8-mediated benefits in antigen recognition are removed by DT227/8KA substitution of HLA-A2 6, suggesting that the role of the human pMHCI/CD8 interaction might be more significant than the effects of direct TCR/CD8 interaction. Our previous findings indicate that the binding energy provided by the external pMHCI/CD8 interaction is subordinate to other roles of this interaction in TCR-mediated signal transduction 18.

The findings of this study pose a number of interesting dilemmas. First, if slightly improving the pMHCI/CD8 interaction can enhance the recognition of peptide antigens, then why has the human immune system not evolved to incorporate this feature? Evidence that different human classical MHCI molecules differ in their affinity for CD8 further compounds the problem 41. Second, within a given CTL clone, we observe a distinctly non-linear relationship between the 3D affinity of the pMHCI/CD8 interaction and its biological role in antigen recognition. It is perplexing that the five-fold reduction in CD8 binding afforded by the A245V substitution can have a minimal negative effect on antigen recognition 18 while a 50% increase in binding confers such a positive effect.

The answer to both these dilemmas may lie in the importance of the kinetics of pMHCI/CD8 binding when compared to TCR/pMHCI interactions as set by thymic selection. The relationship between 3D affinity and the functional consequences of two-dimensional, membrane-constrained, interactions at the lymphocyte cell surface is known to be complex 42 and is not well understood. These complexities may be further enhanced for CD8 18 which is known to have several distinct roles in T cell activation 2–4. Here, we find that these intricacies are open to subtle exploitation by showing that even a small increase in the interaction between human pMHCI and CD8 can result in a substantial improvement in the recognition of MHCI-restricted antigen by cognate CTL. The enhancement of CD8 binding conferred by the Q115E substitution in HLA-A2 results in only a minor difference in TCR/pMHCI off rate and half-life at the cell surface 9 and does not result in significant loss of antigen specificity (Fig. 7).

Human TCR often bind to cognate pMHCI with 10–100-fold higher affinities than human CD8. Murine CD8 binds to murine pMHCI with a substantially higher affinity than the equivalent human interaction 6, such that CD8 might make a more significant contribution to stabilisation of the TCR/pMHCI interaction in this system. Enhancing the already over four-fold stronger murine pMHCI/CD8 interaction still further might lead to a significant loss in antigen specificity. This appears to be the case when we super-enhance HLA-A2/CD8 interactions (K_D_ ∼10 μM) (Laugel *et al.* and Wooldridge *et al*., manuscripts in preparation). Such enhancements bring the pMHCI/CD8 interaction into an affinity range that might allow the initiation of biological effects in its own right 42. Thus, our findings will be difficult to study directly *in vivo* with murine models.

We have shown that Q115E substitution of HLA-A2, which increases the affinity for CD8 from K_D_ ∼130 μM to K_D_ ∼85 μM without altering interactions with the TCR 9, affords enhanced biological recognition of cognate antigen by different CTL with a wide range of antigen specificities. We have recently demonstrated that the Q115E substitution affords a <2% decrease in TCR/pMHCI disscociation at the cell when compared to wild-type MHCI 9. In addition, we have also demonstrated that the pMHCI/CD8 interaction can act to enhance the TCR/pMHCI association rate at the cell surface (Van den Berg *et al*., submitted). This is consistent with the findings of a recent publication 43. The kinetic advantage afforded by the Q115E mutation in terms of TCR/pMHCI binding is likely to enhance antigenicity *per se.* However, we have previously demonstrated that the dominant role of the pMHCI/CD8 interaction is in the recruitment of signaling molecules to the TCR/CD3 complex and not the binding energy that it provides; therefore, this difference is unlikely to account for the extent of the enhancement we observe.

Indeed, most of the improvements in antigenicity with Q115E-substituted MHCI appear to be the result of enhanced early intracellular signal transduction. The molecular mechanisms that underlie the observed enhanced early signal transduction are likely to be complicated as they may be due to either (1) more efficient signalling molecule recruitment to the TCR/CD3 complex or (2) more efficient recruitment of TCR/pMHCI complexes to lipid rafts or other roles of CD8. Importantly, enhancement of CD8 binding appears to augment the recognition of agonist and weak agonist ligands without leading to recognition of ligands with a shorter TCR/pMHCI interaction. Furthermore, CD8 enhancement can be engineered into any human MHCI molecule and, therefore, any MHCI-restricted antigen. CD8-enhanced pMHCI ligands are recognized at lower concentrations, induce significantly more cytokine production, enhance CTL proliferation and are better at priming human CTL directly *ex vivo.* These findings indicate a novel mechanism that could enable boosting of specific cytotoxic immunity, an approach that might be especially pertinent in the context of anti-tumour CTL responses. In addition, the mutations that we describe might be useful in the setting of vaccination strategies to enhance the generation of specific pMHCI-restricted CTL responses.

## Materials and methods

### MHCI transfectants and manufacture of target cells

Mutations in HLA-A2 and the biophysical validation of their effects are published elsewhere 9, 18. Cells transfected with HLA-A2 and mutants thereof were produced as described previously 6. In each case, cells were cloned and tested with relevant antibodies to ensure that they expressed identical levels of MHCI on their surface. The GFP-HLA-A2 fusion vectors were made as described in Fig. 4.

### CTL priming

C1R-A2 cells were pulsed with 1 μM Melan A_26–35_ (ELAGIGILTV) peptide for 90 min, irradiated and washed once in RPMI 1640 medium. Pulsed irradiated C1R-A2 cells (2×10^5^) were incubated with 10^6^ fresh HLA-A2^+^ human PBMC in RPMI 1640 supplemented with 10% fetal calf serum, 100 U/mL penicillin, 100 μg/mL streptomycin and 2 mM glutamine (Sigma) (R10 medium); 200 U/mL IL-2 was added on day 3. Melan A-specific cells were quantified on day 10 with wild-type HLA-A2-ELAGIGILTV tetramer.

### CTL proliferation

Clone ILA1, specific for the human telomerase reverse transcriptase-derived peptide hTERT_540–548_, and clone IC2, specific for hTERT_865–873_, were labelled at 10^6^ cells/mL with 5 μM CFSE dye for 8 min at room temperature. Fetal calf serum was added in excess to stop the reaction. Cells were washed three times and resuspended in R10 medium. CTL (0.5×10^6^) were mixed at an E:T ratio of 1:1 with C1R target cells that had been transfected with the wild-type or the indicated mutant of HLA-A2 and pulsed with 10^–6^ M of hTERT_540–548_, hTERT_865–873_ or EBV (negative control) peptides. Cells were incubated at 37°C in R10 medium supplemented with IL-2 at 50 U/mL final concentration in a 2-mL final volume. Samples were collected on a FACSCalibur flow cytometer and data were analysed with CellQuest software (BD Biosciences). A minimum of 10 000 live cells were analysed per sample.

### Surface plasmon resonance, soluble pMHCI and TCR manufacture, and CTL activation assays

Soluble TCR and pMHCI manufacture, tetramerization and biophysical studies were performed as previously described 9. Preparations of pMHCI tetramer were shown to be >98% tetrameric (data not shown). CTL culture in IL-2 or IL-15 and functional bioassays are also described elsewhere 10, 18.

### Antiphosphotyrosine immunoblots

Antigen-specific CTL (10^6^) were exposed to peptide-pulsed C1R targets at an E:T ratio of 1:10 for 10 min, or 10 μg/mL tetramer for 3 min, then lysed on ice for 30 min. The nuclear fraction was pelleted by centrifugation, the remaining lysate aspirated, added to an equal volume of SDS loading buffer (350 mM Tris pH 6.8, 350 mM SDS, 30% glycerol, 600 mM DTT, 175 µM Bromophenol Blue) and boiled for 6 min. Samples were then loaded into a 12% SDS-PAGE protein gel for electrophoresis at 100 V for 16 h. Protein was transferred from the gel by electrophoresis at 25 V for 50 min. After blocking for 1 h (1% BSA), the membrane was incubated with 1 μg/mL mouse anti-phosphotyrosine antibody (clone 4G10; Upstate Biotechnology) for 4 h. After washing, the membrane was incubated with 0.25 μg/mL sheep anti-mouse peroxidase-linked secondary antibody (Amersham) in 1% BSA, 2.5% milk powder for 1.5 h. The blot was washed, then developed using chemiluminescent substrate Supersignal Pico (Perbio). Data were collected using a Bio-Rad Fluor-S™ Multimager.

## Abbreviations:

MHCI: MHC class I
pMHCI: peptide-MHCI complex
SPR: surface plasmon resonance
